# Key considerations for combination therapy in Alzheimer's clinical trials: Perspectives from an expert advisory board convened by the Alzheimer's drug discovery foundation

**DOI:** 10.1016/j.tjpad.2024.100001

**Published:** 2025-01-01

**Authors:** Jeffrey Cummings, Michael Gold, Mark Mintun, Michael Irizarry, Andrew von Eschenbach, Suzanne Hendrix, Donald Berry, Cristina Sampaio, Kaycee Sink, Jaren Landen, Miia Kivipelto, Michael Grundman, Steven E. Arnold, Allan Green, Katherine Partrick, Laura Nisenbaum, Aaron Burstein, Howard Fillit

**Affiliations:** a Chambers-Grundy Center for Transformative Neuroscience, Department of Brain Health, School of Integrated Health Sciences, University of Nevada, Las Vegas, Las Vegas, NV, USA; b Compass Pathways, London, UK; c Eli Lilly and Company, Indianapolis, IN, USA; d Eisai Inc, Nutley, NJ, USA; e Samaritan Health Initiatives, Houston, TX, USA; f Pentara Corporation, Millcreek, UT, USA; g Department of Biostatistics, The University of Texas MD Anderson Cancer Center, Houston, TX, USA, Berry Consultants, LLC, Austin TX, USA; h CHDI Management, Inc. the company that manages the scientific activities of CHDI Foundation Princeton, NJ, USA, Faculdade Medicina University of Lisbon, Lisboa, Portugal; i Genentech Inc., South San Francisco, CA, USA; j Biogen, Cambridge, MA, USA at the time of advisory board; k Department of Neurobiology, Care Sciences and Society, Center for Alzheimer Research, Karolinska Institutet, Stockholm, Sweden, Ageing Epidemiology Research Unit (AGE), School of Public Health, Faculty of Medicine, Imperial College London, UK, Institute of Public Health and Clinical Nutrition, University of Eastern Finland, Kuopio, Finland; l Global R&D Partners, LLC., San Diego, CA, USA and University of California, San Diego, San Diego, CA, USA; m Department of Neurology, Harvard Medical School, Massachusetts General Hospital, Boston, MA, USA; n SDG, LLC, Cambridge, MA, USA; o Independent Researcher, Albuquerque, NM, USA; p Alzheimer's Drug Discovery Foundation, New York, NY, USA

**Keywords:** Alzheimer's disease, Combination therapy, Clinical trials, Preclinical research, Biomarkers

## Abstract

There is growing consensus in the Alzheimer's community that combination therapy will be needed to maximize therapeutic benefits through the course of the disease. However, combination therapy raises complex questions and decisions for study sponsors, from preclinical research through clinical trial design to regulatory, statistical, and operational considerations. In January 2024, the Alzheimer's Drug Discovery Foundation convened an expert advisory board to discuss the key considerations in each of these areas. Experts agreed on the need to prioritize a combination therapy approach that encompasses a wide range of targets associated with aging and the underlying biology of Alzheimer's disease. Progress in combination therapy could be accelerated by leveraging preclinical research and Phase 1 and 2A trials to identify the most promising combinations for further development, exploring repurposed agents with available preclinical and clinical data, building collaborations across sectors to support operational challenges, and planning for the likely impact of anti-amyloid beta-protein monoclonal antibody therapies on future clinical trial designs.

## 1 Introduction

There is a growing consensus in the Alzheimer's community that combination therapy will be needed to maximize therapeutic effect across the continuum of disease. This consensus is driven by the growing understanding of the diverse, interconnected mechanisms underlying Alzheimer's disease [[1], [2], [3]]; the success of combination therapy for other complex chronic diseases like cancer and heart disease; and the modest effects on decline produced by the new generation of monoclonal antibodies targeting β-amyloid. Like many other chronic diseases, aging is the leading risk factor for Alzheimer's, and various aging-related processes have been implicated in the development and progression of the disease [1]. A comprehensive treatment approach that encompasses the multiple mechanisms of pathogenesis will be necessary to address Alzheimer's on multiple fronts.

Combination therapy raises complex questions and decisions for sponsors, from preclinical research through clinical trial design to regulatory, statistical, and operational considerations. Previous expert convenings and publications have highlighted the importance of these questions for the future of Alzheimer's treatment [4,5]; given recent advances in the field, now is the time to begin more concerted, urgent, and collaborative engagement with these questions to drive progress towards delivering combination therapies.

In January 2024, the Alzheimer's Drug Discovery Foundation (ADDF) convened a panel of experts from the research, industry, clinical, and regulatory communities to discuss the key considerations that will guide the path forward for combination therapy. There was consensus on the following priority action areas:

• The Alzheimer's field has reached a critical juncture where new therapies are advancing in the pipeline and coming to market, creating a need to prioritize a combination therapy approach, especially for a wider range of non-amyloid targets that reflect the multifactorial nature of the disease. There are multiple combination therapies in the current Alzheimer's disease pipeline [6], and it is expected that the number of combination therapy trials will grow in the years ahead. Given the complexity of studying and managing combination therapies, sponsors must begin now to plan, collaborate, and advance this area of research.
• There is an important role for preclinical research in the efficient development of combination therapies. Preclinical studies can identify mechanisms of action with potential synergistic or additive effects, identify new biomarkers, and provide early data regarding safety, pharmacokinetics, and pharmacodynamics.
• Phase 1 and 2A clinical trials of combination therapies are essential to lay the groundwork for later-stage trials. Phase 1 trials are important to understand dose, safety, and tolerability; dose-response data, dose-limiting toxicity, and pharmacokinetics must be understood for each new agent and for the combination. Iterative Phase 2A hypothesis-generating trials will likely be necessary to inform the design of later-stage trials and to identify the most promising combinations warranting additional investment. Moreover, innovations in clinical trial designs should be considered to support the efficiency of combination therapy development.
• Repurposed agents offer one ideal starting point for combination therapy in Alzheimer's disease. These agents are often more accessible and have the advantage of available preclinical and clinical data that can be leveraged, potentially accelerating clinical development and regulatory pathways for approval (e.g., FDA 505(b)(2) pathway). The number of repurposed agents in the Alzheimer's disease pipeline has increased in recent years, making up 31% of the research pipeline [6]. Those targeting aging-related mechanisms will likely have the greatest promise.
• Collaboration across sectors will be needed to advance combination therapy. Close engagement with regulators will be critical. Momentum can be created with platform trials, non-competitive consortia, and collaboration among biopharmaceutical companies, biotech companies, and not-for-profit entities to navigate the operational, legal, and strategic complexity presented by combination therapies.
• The potential impact of recently approved anti-amyloid beta-protein (Aβ) monoclonal antibody therapies on future clinical trials for combination therapies warrants further dedicated discussion and consideration.

A summary of the advisory board's key considerations and key questions is provided below, as well as a high-level perspective on the state of the field, landscape for combination therapy, and lessons from combination drug development in oncology.

## 2 State of the field

In the 5+ years since previous expert convenings on combination therapy in Alzheimer's disease, including a panel at the 2018 Alzheimer's Association Research Roundtable and a meeting of the European Union/United States/Clinical Trials in Alzheimer's Disease (EU/US/CTAD) task force [4,5], understanding of the underlying biology, therapeutic targets, and new biomarkers for diagnosis and demonstration of target engagement has significantly expanded. Critically, anti-Aβ monoclonal antibody therapies have shown positive results in Phase 3 clinical trials [7,8], resulting in Food and Drug Administration (FDA) approval of two agents (Leqembi and Kisunla) [9,10]. However, anti-Aβ therapies, alone, are likely insufficient to completely stop disease progression, given the complexity of the underlying pathogenic pathways.

These scientific advances indicate the need to pursue a wider range of non-amyloid targets associated with aging and the underlying biology of Alzheimer's disease, such as inflammation, vascular dysfunction, aberrant proteostasis and autophagy, tau protein abnormalities, mitochondrial oxidative stress, metabolic dysfunction, cellular senescence, and epigenetic dysregulation [1,2,11]. There are promising therapeutics in development that target these processes, indicating the potential for combination therapies that address multiple dysregulated systems with additive or synergistic effects.

This approach aligns with the overall state of the Alzheimer's disease therapeutic pipeline, as over 70% of disease-modifying therapies in the 2024 pipeline target biology other than amyloid or tau [6]. The pipeline also includes several combination therapies, though the number is currently limited. Additionally, the share of repurposed agents in the pipeline continues to grow – providing a promising starting point for combination therapy.

### 2.1 Understanding the landscape for combination therapy & applying lessons from oncology

While the rationale for combination therapy is clear, the path forward is complex. As sponsors turn to this area, they must navigate a set of interrelated questions, decisions, and potential development avenues. These include:

• Different types of combination therapies, such as combination products, a sequential combination, add-on therapies, and multi-target drugs.
• The regulatory implications of study design, dose optimization, and endpoint acceptability of two experimental agents developed together, one approved and one experimental agent developed together, or the use of approved or experimental therapies as add-on treatment to anti-Aβ monoclonal antibodies or other clinically available therapies.
• Different modalities in combination (e.g., small molecule therapies, biologic therapies, medical devices, and lifestyle interventions) and/or different formulations (e.g., oral, intravenous, intramuscular, intrathecal, subcutaneous, transdermal, or intra-nasal).
• Different rationales for pursuing a combination therapy, including improved efficacy, use of multiple mechanisms of action, improvement in pharmacokinetics, and targeting of specific clinical symptoms which develop as the disease progresses.
• Different patient needs in a heterogenous disease, as well as different combination strategies across the continuum of disease, from preventative or early interventions through interventions later in disease course.
• Alterations in the interpretation of biomarkers, especially given the rising number of patients with varying exposures (dose and duration) to anti-Aβ monoclonal antibodies.

While these considerations introduce complexity, other therapeutic areas, principally oncology, have shown that progress with combination therapy is not only possible, but likely necessary for effective treatment. The expert advisory board highlighted four key lessons from oncology for Alzheimer's disease: the importance of understanding the underlying biology and interactions among diverse mechanisms of disease; the potential for repurposed drugs, including previously shelved therapies, to provide impactful effects in combination; the necessity of considering adaptive trial designs and discussing them with the FDA early in the process; and the need for integrated, interdisciplinary solutions, developed through collaboration within the field and with other fields, using interoperable data systems to deliver therapies for patients.

The Alzheimer's field needs a similar approach. To aid sponsors in achieving this goal, we summarize considerations and questions drawn from the advisory board. These are intended to provide a framework that can inform discussions, generate ideas, and support sponsors as they make decisions based on their specific goals.

## 3 Regulatory considerations

The FDA provides guidance for development of combination products which varies depending on the type of combination [12]. No equivalent guidance is currently available from the European Medicines Agency (EMA). Advisory board members agreed that the Alzheimer's field needs to be educated not only on the FDA guidance, but also what the framework indicates about the overall regulatory perspective on combination therapy.

### 3.1 Key considerations

*Sponsors can consider the following areas for engaging regulators on combination therapy:*

The Alzheimer's field overall must use consistent language and definitions aligned with the FDA in regard to combination therapy. This consistency is essential, given that the FDA guidelines vary depending on the type of combination therapy. Similar recommendations from the EMA underscore the importance of considering analogous regulatory interactions with authorities globally.

The experience of other disease areas shows the value of engaging the FDA early, with Pre-Investigational New Drug (p-IND) meetings to work through questions and decisions. Through the Prescription Drug User Fee Act (PDUFA), the FDA must provide pre-IND advice to study sponsors. Most issues that result in IND refusals to file or clinical holds can be avoided with pre-IND meetings. Designing preclinical and Exploratory IND Phase 0 studies to evaluate pharmacokinetics and target engagement helps build confidence that there is a strong rationale for combination therapy, based on data and a good understanding of toxicity.

### 3.2 Key questions

*Sponsors can consider the following regulatory questions:*

• How can the data collected in the preclinical phase and early-stage trials be maximized to build confidence among regulatory authorities in the rationale for exploring combination therapy and accelerate clinical development?
• What patient inclusion and exclusion criteria and clinical study endpoints help ensure that study data in combination drug trials will support NDA approval for the targeted indication for use?
• What are the unique regulatory considerations for different types of combinations?
• Are there unique safety concerns arising from the combination therapy that must be addressed?

## 4 Considerations for the impact of anti-aβ monoclonal antibody therapies on trial design

The FDA's approval of two anti-Aβ monoclonal antibody therapies (Leqembi and Kisunla) make it likely that a growing number of patients with early Alzheimer's disease will be exposed to anti-Aβ monoclonal antibody therapies [9,10]. This class may become part of the standard of care for eligible patients in the future, in which case the complexity of trials for combination therapy may increase dramatically. As sponsors consider how these therapies should be included, the drug under investigation, objectives of the trial, disease stage, and safety risks will guide how inclusion of participants on anti-Aβ monoclonal antibodies affects the clinical development program.

These questions and considerations related to anti-Aβ monoclonal antibodies and their impact on the future of clinical trials will need to be considered. Greater collaboration and prioritization are needed to ensure the field is prepared for the implications of these developments.

### 4.1 Key considerations

*Sponsors can consider the following areas related to the inclusion of anti-Aβ monoclonal antibody therapies in combination trials:*

First, sponsors will need to decide whether to allow anti-Aβ monoclonal antibodies into a combination therapy trial as prior medication, background therapy, or cotreatment. Incorporating anti-Aβ monoclonal antibodies into clinical trials would require that participants meet eligibility criteria for any anti-Aβ monoclonal antibodies allowed, while ensuring the sample size is sufficiently large for adequately powered analyses. If anti-Aβ monoclonal antibodies are included as cotreatment, study sponsors will need to decide which monoclonal therapy, as well as the appropriate dosing regimen and safety monitoring plan for the combination study. To reduce potential confounding and safety issues, sponsors may also want to consider whether doses of a previously administered anti-amyloid monoclonal antibody should be stable for a certain length of time; or if discontinued, discontinued for a certain length of time before allowing subjects to be randomized in a clinical trial of a new investigational agent.

Further, anti-Aβ monoclonal antibody exposure will have implications for the interpretation of biomarkers. For example, treatment-related amyloid clearance may mean patients no longer meet amyloid positron emission tomography criteria for Alzheimer's disease diagnosis, and the biomarker changes of a novel agent will occur on top of significant existing changes. The Alzheimer's Association's Treatment-Related Amyloid Clearance (TRAC) Working Group is currently examining this area. In addition to amyloid plaque removal, reductions in plasma phosphorylated-tau (e.g., p-tau 181 and p-tau 217) as well as decreased glial fibrillary acidic protein are observed in patients receiving treatment with anti-Aβ monoclonal antibodies, making interpretation of these biomarkers more difficult in combination and add-on trials.

### 4.2 Key questions

*Sponsors can consider the following questions related to anti-Aβ monoclonal antibody therapies:*

• Will anti-Aβ monoclonal antibodies become standard of care for early Alzheimer's disease? If so, when is this likely to happen, and what are the implications on combination trials? Will there be demographic and geographical differences?
• What are the trial design implications for anti-Aβ monoclonal antibodies as background therapy; for anti-Aβ monoclonal antibodies as a combination trial agent?
• How will sponsors and trial investigators monitor amyloid-related imaging abnormalities and identify potential synergistic toxicities when investigating novel agents as add-on therapy to an anti-Aβ monoclonal antibody?
• How should changes in the interpretation of biomarkers related to anti-Aβ monoclonal antibodies be approached?

## 5 Trial design considerations

Combining novel therapies with approved treatments can be investigated using an add-on therapy design that compares the combination to the novel agent alone. When studying combinations of two or more investigational agents, innovative trial designs may represent an opportunity to efficiently study these agents in combination. Factorial or modified factorial designs may be needed to demonstrate the contribution of each therapeutic agent to the combined effect and to characterize whether the effect is additive, synergistic, or antagonistic. Advancements in biomarker development require that sponsors consider how to best leverage novel biomarkers in combination trial designs. Additionally, sponsors will need to strategize on integrating validated measures of clinical benefit to comprehensively assess therapeutic effects, particularly in later-stage trials.

### 5.1 Key considerations

*Sponsors can consider the following areas for innovative trial design:*

Preclinical research studies should be designed to examine combined mechanisms and identify synergistic, additive, or antagonistic effects. In addition, preliminary modeling of pharmacokinetics and pharmacodynamics for treatment interaction can reduce risk of failure.

Phase 1 and Phase 2 trials should be designed to identify early safety signals, inform dosing strategy, and provide insight into the clinical effect of the combination. Optimal dosing, including dose-limiting toxicity, should be well-understood in the early stages of development to meet regulatory requirements for a successful Phase 3 trial. In pivotal Phase 3 trials, study sponsors must consider the clinical outcomes that will best reflect meaningful benefits of the combination. FDA New Drug Application approval requires the demonstration of clinical benefit, not just biomarker changes. Further, statistical study design will be important to determine appropriate sample sizes that will be needed as the safety and efficacy of each agent in the combination may require separate evaluation and comparison with the combined effect.

Additionally, early regulatory approval may be obtained with rigorous post-market surveillance. Phase 4 studies and real-world evidence can evaluate the persistence of the effects of combination therapy, the need for dose adjustments, data from under-studied populations, and long-term safety and tolerability.

Sponsors will also need to consider biomarker strategy for every step of the development program, from recruitment through clinical effect and safety monitoring. Biomarkers should be leveraged to inform participant selection, as well as target engagement and the pharmacodynamics of each agent in combination, and to support the pathophysiological and clinical effect of the combination. For the field overall, it is important to align and clearly define terminology for biomarker analysis and how the terminology changes in combination drug development.

### 5.2 Key questions

*Sponsors can consider the following trial design questions:*

• What mechanisms are involved, and to what extent at a given state in the disease process?
• What will the dosing schedule be? (Combinations that pose difficult adherence challenges for participants should be avoided.)
• How to navigate participant heterogeneity, particularly due to differing exposures to anti-Aβ monoclonal antibodies?
• What degree of clinical and biomarker change is clinically meaningful and, ultimately, meaningful for regulators and valued by payers?
• How to account for changes in the fast-evolving field? (e.g., availability of blood-based biomarkers, global approval of anti-Aβ monoclonal antibodies, and establishment of these therapies as standard of care)
• How will a deeper understanding of each patient's biology with biomarkers impact trial design? For example, how will such data guide participant selection and the formation of treatment groups? Should participants with synucleinopathies, for instance, be considered as a distinct treatment group?

## 6 Statistical considerations

Consideration should be given to how to power studies with a sufficient, but still achievable sample size, and to navigate heterogeneity and patient-patient variability. Covariates and risk models can help to address this variability. Employing Bayesian statistical methods and adaptive trial designs is an important approach, and FDA guidelines for such approaches are available [13].

Large sample sizes may be needed for some trials of combination therapy, especially those that target early disease. For example, a 4-arm classic 2 × 2 factorial design may require a larger sample size. Achieving the required sample size is also impacted by the patient population, experience with background therapy, the type of combination, whether effects are additive, synergistic, or antagonistic, and the rate of disease progression.

Adaptive trial designs and a Bayesian statistical approach can allow better assessment of efficiency of trials, with faster time to conclusions [14]. However, the FDA must be engaged early to ensure regulatory alignment with adaptive trial designs.

### 6.1 Key questions

*Sponsors can consider the following questions regarding statistical implications:*

• What is the target patient population, disease stage, and number of comparisons needed for the combination, and how does this impact the sample size required for sufficient statistical power?
• What adaptive study designs or alternative approaches could be discussed with regulatory authorities? When should these conversations be held?
• What endpoints and statistical criteria will be sufficient to demonstrate contribution of components?

## 7 Operational and strategic considerations

Combination therapy with two or more investigational agents may present significant operational and strategic challenges, especially if different sponsors are combining agents. These include cross-licensing of patents, agreements about who will manufacture and distribute the combination therapy, and the mechanisms to reach agreement on clinical trial design, validated biomarkers and biomarker analysis, dose selection, safety monitoring, shared intellectual property, data ownership, and more.

While these challenges can be solved, the field must begin to engage with these questions promptly. Biopharmaceutical companies would potentially require multiple years to work through these questions prior to entering formal partnership agreements. Larger biopharmaceutical companies may have concerns about such agreements, given that risk-benefit cannot be predicted, safety may be the responsibility of multiple entities, and incentives may be limited. A combination of two novel agents from two biopharmaceutical companies may make these challenges more difficult.

### 7.1 Key considerations

*Sponsors can consider the following areas for implementing combination therapy approaches:*

Sponsors can consider models that mitigate competing commercial incentives, such as a non-competitive consortium or a collaboration between a biopharmaceutical company and a not-for-profit entity, an academic institution, or a public-sector entity. Sponsors might also consider a platform trial with a precompetitive process, where the IND application is led by a not-for-profit entity.

For collaboration involving a for-profit company, potential incentives could include co-funding, potential for intellectual property expansion, differentiation within a therapeutic class, support for pricing negotiations, label extension, and increased likelihood of commercial success if a monotherapy is unlikely to succeed. There is also potential for larger biopharmaceutical companies to leverage their open-label extension to test novel drugs in combination with well-studied assets, as well as for those investigating anti-Aβ monoclonal antibodies to conduct smaller sub-studies that examine a novel agent with the antibody as background therapy.

Given operational and strategic challenges, repurposed drugs offer sponsors a potential starting point for developing combination therapy. There are many opportunities to repurpose agents to support or accelerate combination therapy in Alzheimer's disease, with those indicated for other age-related neurological disease holding the greatest promise.

In addition, a consortium can be built to provide guidelines on conducting combination trials. Similar models have been implemented in rheumatology, pain, and stroke treatment development programs. In the Alzheimer's field, the Dominantly Inherited Alzheimer Network Trials Unit (DIAN‐TU) platform is an important example of collaboration, including the Tau NexGen trial that will conduct “the first Alzheimer's prevention trial to target both tau tangles and amyloid plaques with two drugs at the same time.” [15]

### 7.2 Key questions

*Sponsors can consider the following operational and strategic questions:*

• How might sponsors collaborate to merge data from platform trials assessing multiple drugs concurrently? Valuable insights on this approach can be gained from past initiatives [16].
• How can the field collaborate to advance biomarker banking and sampling to support combination trials?
• How should data ownership be determined? What are best practices for mutual disclosure of results?
• How can repurposed therapies be leveraged to mitigate the challenges of testing two novel compounds in combination?

## 8 Conclusion

The Alzheimer's field is positioned for the development of combination therapies. The combination approach is supported by the need to target multiple pathophysiologic pathways implicated in Alzheimer's and aging, preclinical models that provide scientific rationale, and advances in biomarker development and clinical outcomes to assess the contribution of different agents in combination. Combination trials will pave the way for precision medicine in Alzheimer's, where combination therapies can be tailored to a patient's individual profile.

While combination therapy presents complex considerations and challenges, other disease areas illustrate that these challenges can be overcome, especially by employing innovative models and collaborations. Given the projected increases in prevalence and costs of Alzheimer's disease, the field must move with urgency to lay the groundwork for these collaborations, consider the likely impact of anti-Aβ monoclonal antibodies on the future of trial design, and ultimately develop the right models to advance a range of different combination therapies for the future of Alzheimer's disease care.

## Declaration of interests

JC has provided consultation to Acadia, Acumen, ALZpath, Annovis, Aprinoia, Artery, Biogen, Biohaven, BioXcel, Bristol-Myers Squib, Eisai, Fosun, GAP Foundation, Green Valley, Janssen, Karuna, Kinoxis, Lighthouse, Lilly, Lundbeck, LSP/eqt, Merck, MoCA Cognition, New Amsterdam, Novo Nordisk, Optoceutics, Otsuka, Oxford Brain Diagnostics, Praxis, Prothena, ReMYND, Roche, Scottish Brain Sciences, Signant Health, Simcere, sinaptica, TrueBinding, and Vaxxinity pharmaceutical, assessment, and investment companies. JC owns the copyright of the Neuropsychiatric Inventory. JC has stocks/options in Artery, Vaxxinity, Behrens, Alzheon, MedAvante-Prophase, Acumen. JC is supported by 10.13039/100000057NIGMS grant P20GM109025; NIA grant R35AG71476; NIA R25 AG083721-01; Alzheimer's Disease Drug Discovery Foundation (ADDF); Ted and Maria Quirk Endowment; Joy Chambers-Grundy Endowment. AG is external counsel for the ADDF and a member of the ADDF Board of Overseers; AG has no financial interest in the materials or related companies discussed in this manuscript. MG(b) is an employee of Global R&D Partners, LLC. JL was a Biogen colleague when this panel was conducted and is a shareholder. AE holds Board of Director positions on Bausch and Lomb, Trisalus Biosciences and the Reagan Udall Foundation. MCI is an employee of Eisai, Inc. KC is a full-time employee of Genentech, Inc., a member of the Roche Group, and a shareholder in F. Hoffmann-La Roche. CS has received consulting fees from Novartis, Biogen, and Eisai. MK is on the scientific advisory boards for Combinostics, BioArtic, Eisai, Eli Lilly, and Nestle, has served as a speaker for Eisai, Nestle, Nutricia, and Novo Nordisk, is the Director of R&D, Karolinska University Hospital, and receives grant support from 10.13039/501100002341Academy of Finland, Swedish Research Council, Alzheimer's Research and Prevention Foundation, EU 7th framework, CIMED, JPND, IMI, Wallenberg Clinical grant, FORTE, KI-Janssen Strategic Collaboration, Imperial College ITMAT, Innovative Health Initiative (IHI), Gates Ventures, ADDI, Alzheimer's Drug Discovery Foundation, Part the Cloud. DB is co-owner of Berry Consultants, LLC, a company that designs adaptive Bayesian clinical trials for pharmaceutic and medical device companies, NIH cooperative groups, patient advocacy groups, and international consortia. SH is owner of Pentara Corporation, who performs statistical consulting services for dozens of companies in the Neurodegenerative space. SEA has received grants or contracts from AbbVie, AC Immune, Alzheimer's Association, Alzheimer's Drug Discovery Foundation, Amylyx, Athira, Challenger Foundation, Chromadex, EIP Pharma, Ionis Pharmaceuticals, Janssen Pharmaceuticals, John Sperling Foundation, National Institutes of Health, Novartis, and Seer Biosciences, and has provided consultation to BioVie, Daewoong Pharmaceutical, EIP Pharma, and Risen Pharmaceutical Technology. SEA has received payment for testimony from Boyle Shaughnessy Law, is a co-inventor on a pending patent application related to assays for neurological biomarkers, and on advisory boards for Allyx Therapeutics, Bob's Last Marathon, Cortexyme/Quince, Jocasta Neuroscience, Sage Therapeutics, Sanofi, Vandria, and BioVie. HF has consulted with Alector, LifeWorx, Mediflix, Samus Therapeutics, Otsuka Pharmaceuticals, Pinteon Therapeutics and has served as an unpaid consultant for Eli Lilly. MG(a), KP, LN and AB have no conflicts of interest to disclose.
